# Supplementary material for: Altitudinal Variation Influences Soil Fungal Community Composition and Diversity in Alpine–Gorge Region on the Eastern Qinghai–Tibetan Plateau
Source: J Fungi (Basel). 2022 Jul 30;8(8):807. doi: 10.3390/jof8080807 (PMC9410234; doi:10.3390/jof8080807)
Supplement: Supplementary file 1 [file jof-08-00807-s001.zip › jof-1811026-supplementary.pdf]

Supplementary Material

Altitudinal Variation Influences Soil Fungal Community Composition and Diversity in Alpine-Gorge Region on the Eastern Qinghai-Tibetan Plateau

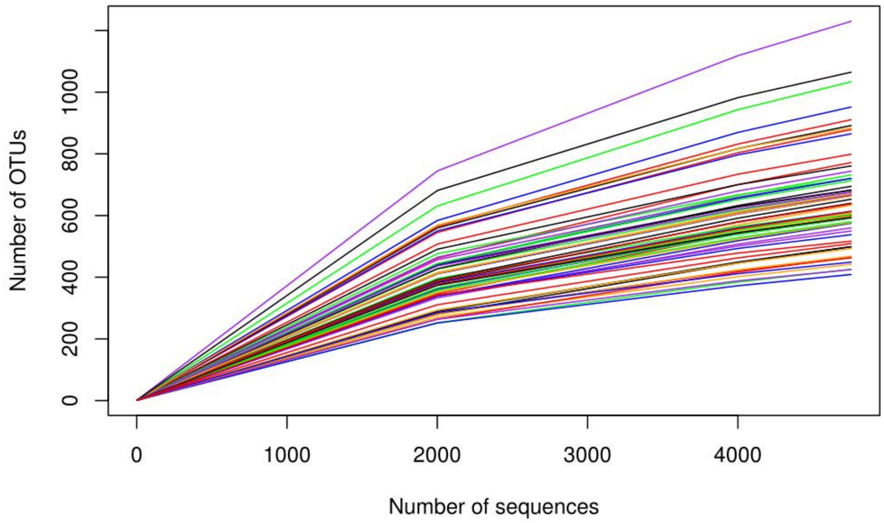

Figure S1. Rarefaction curve showing the sequence depth and observed OTUs.
